# Buzhong Yiqi Decoction Regulates Th17 Cells Differentiation in Autoimmune Thyroiditis through PTEN/AKT-immunometabolism Axis

**DOI:** 10.2174/0118715303388096250914111152

**Published:** 2025-10-15

**Authors:** Jiayun Li, Zhuo Zhao, Donglin Liu, Yue Luo, Feng-yi Guo, Zhi-min Wang, Nan Song, Ziyu Liu, Xiao Yang

**Affiliations:** 1 The Second Clinic College, Liaoning University of Traditional Chinese Medicine, Shenyang, Liaoning 110847, China;; 2 The Second Affiliated Hospital of Liaoning University of Traditional Chinese Medicine, Shenyang, Liaoning 110034, China;; 3 Department of Endcocrinology, Affiliated Hospital of Liaoning University of Traditional Chinese Medicine, Shenyang, Liaoning110032, China;; 4 Liaoning Institute of Traditional Chinese Medicine, Shenyang, Liaoning 110034, China

**Keywords:** Buzhong yiqi decoction, autoimmune thyroiditis, immunometabolism, T helper cell 17, PTEN/AKT signaling pathway, hypothyroidism

## Abstract

**Introduction:**

Autoimmune thyroiditis (AIT), a prevalent autoimmune disorder, can severely impair patients' quality of life when progressing to hypothyroidism. This investigation seeks to systematically evaluate the therapeutic efficacy of Buzhong Yiqi Decoction (BZYQ) against iodine-induced AIT while elucidating its underlying molecular mechanisms.

**Methods:**

NOD.H-2^h4^ mice received daily 0.05% sodium iodide gavage for 8 weeks to induce AIT. Thyroid histopathology was evaluated by HE staining. Serum autoantibody levels and lactate concentrations were measured. Western blotting analyzed PTEN, p-PI3K/PI3K, p-AKT/AKT, GLUT1, HK2, PKM2, and LDHA protein expression in thyroid tissues. Immunofluorescence staining localized the expression of HK2 and RORγt in thyroid sections. Flow cytometry quantified the proportion of Th17 cells within splenic lymphocytes.

**Results:**

BZYQ reduced thyroid structural damage and lymphocytic infiltration in AIT mice, accompanied by significant reductions in serum TGAb and TPOAb levels. Notably, BZYQ modulated glycolysis to decrease lactate levels and the proportion of Th17 cells. Mechanistically, BZYQ upregulated PTEN expression to suppress downstream p-PI3K, p-AKT, GLUT1, HK2, PKM2, and LDHA expression, thereby achieving regulation of immunometabolic pathways.

**Discussion:**

This study provides experimental evidence for broadening the modern clinical applications of this classical formula. However, the related mechanisms still require further rigorous clinical studies for validation.

**Conclusion:**

BZYQ modulates Th17 differentiation *via* the PTEN/AKT-immunometabolic axis in AIT.

## 1 INTRODUCTION

Autoimmune thyroiditis (AIT) is a common autoimmune disease characterized by immune cell infiltration of the thyroid gland, accompanied by elevated serum levels of thyroid antibodies [1, 2]. Recent epidemiological data indicate that approximately 7.5% of the global population carries AIT-related autoantibodies [3]. As the disease progresses, this immune-mediated damage to the thyroid gland may lead to hypothyroidism, affecting multiple organ systems, including the cardiovascular and nervous systems, thereby increasing overall mortality rates [4, 5]. Currently, clinical management primarily relies on thyroid hormone replacement therapy, anti-inflammatory medications, and symptom-oriented interventions [6-8]. However, no definitive therapeutic strategy has yet been established to effectively restore immune homeostasis and prevent disease progression.

The immune dysregulation in AIT arises from three interconnected factors: genetic susceptibility, environmental triggers, and impaired immune tolerance [9, 10]. Recent advances in immunometabolism highlight bioenergetic reprogramming as a key regulator of immune cell differentiation and effector functions [11]. Specifically, CD4^+^ T cells in AIT patients demonstrate amplified glycolytic flux, which drives T helper 17 (Th17) differentiation *via* aerobic glycolysis [12-14]. In AIT, Th17 cells exacerbate thyroid tissue inflammation and autoimmune responses by secreting proinflammatory cytokines such as interleukin-17 (IL-17). This cytokine cascade leads to thyroid follicular destruction and autoantibody production [15, 16]. Emerging evidence highlights the pivotal role of Th17 transcriptional network dysregulation in autoimmune pathogenesis. Notably, this regulatory aberration has been mechanistically linked to uveitis development, with broader implications across multiple autoimmune pathologies [17]. In Th17-dominated inflammatory microenvironments, upregulated glycolytic activity results in substantial lactate accumulation [18]. This lactate subsequently fuels Th17 differentiation, generating a self-sustaining proinflammatory loop that intensifies autoimmune tissue injury [19]. Accumulating evidence demonstrates that blocking the activation of the phosphatidylinositol 3-kinase/protein kinase B (PI3K/AKT) signaling pathway in AIT effectively suppresses Th17 cell differentiation, thereby alleviating thyroid lymphocytic infiltration and follicular structural destruction [20]. As a core metabolic regulator, Phosphatase and tensin homolog (PTEN) inhibits PI3K *via* dephosphorylation, blocking AKT activation [21]. Emerging evidence indicates that AKT modulates immunocyte energy metabolism through Glucose Transporters (GLUTs), enhancing glucose uptake and utilization to amplify functional activity [22]. Therefore, targeting the PTEN/AKT-immunometabolic axis could inhibit lactate-driven Th17 polarization. This approach may ultimately mitigate thyroid lymphocytic infiltration and tissue damage in AIT.

Buzhong Yiqi Decoction (BZYQ) is a traditional classical Chinese medicinal formula composed of eight herbs. The integration of traditional Chinese medicine principles with empirical evidence demonstrates multifaceted therapeutic effects. Tonification-mediated qi enhancement, coupled with immunomodulatory reinforcement of spleen function, collectively contributes to mitigating immune dysregulation and improving clinical outcomes in AIT. Building upon prior transcriptome profiling studies from our research group, BZYQ has been demonstrated to exert modulatory effects on Th17 cell differentiation pathways [23]. This study elucidates BZYQ-mediated glycolytic modulation *via* the PTEN/AKT signaling pathway during disease progression. The regulatory mechanisms underlying Th17 cell differentiation through immunometabolic control were particularly investigated.

## 2 MATERIALS AND METHODS

### 2.1 Drug Composition and Preparation

BZYQ contains the following components (dosages per formula unit): *Astragalus membranaceus* (Huangqi) 18g, *Atractylodes macrocephala* (Baizhu) 9g, *Glycyrrhiza uralensis* (Zhigancao) 9g, *Panax ginseng* (Renshen) 6g, *Citrus reticulata Blanco* (Chenpi) 6g, *Actaea cimicifuga* (Shengma) 6g, *Bupleurum chinense* (Chaihu) 6g, and *Angelica sinensis (Oliv.)* Diels (Danggui) 3g. All herbal materials were supplied by Anhui Puren Chinese Herbal Medicine Co., Ltd., with quality certification compliant with the Chinese Pharmacopoeia.

The herbal materials underwent immersion in distilled water at an 8:1 (water:herb) ratio for 120 minutes, followed by rapid boiling and subsequent gentle reflux for 60 minutes. Post-extraction, the marc and liquid phase were separated *via* sterile gauze filtration. A secondary extraction was performed by adding fresh solvent, boiling for 30 minutes, and re-filtering. Combined filtrates were concentrated, then cryopreserved at 4°C for downstream applications.

The yeast selenium tablets (Manufacturer: Mudanjiang Lingtai Pharmaceutical Co., Ltd.; Lot: 230305; Approval ID: H10940161) underwent size reduction to fine particles followed by aqueous dispersion using distilled water. The resultant mixture was precisely calibrated to achieve a target concentration of 1.625 μg/mL.

### 2.2 Experimental Animals

NOD.H-2^h4^ mice (Stock No. 004447; Animal Qualification Certificate: 2211A01937) were sourced from Jackson Laboratory. Experimental protocols received ethical approval from Liaoning University of Traditional Chinese Medicine (Approval ID: 21000042023061). All animals were maintained under specific pathogen-free conditions at the University's Experimental Animal Center (License: SYXK (Liao)2019-0004), with environmental parameters controlled at 20-25°C ambient temperature, 50% ± 20% relative humidity, and standardized 12h/12h light-dark cycling.

### 2.3 Animal Model of AIT and Treatments

Forty NOD.H-2^h4^ mice (8-week-old, 20 ± 2 g body weight, equal gender distribution) were randomly assigned *via* a random number table method to four experimental cohorts: Control group (CON), AIT Model group, BZYQ group, and Selenized Yeast group (SYT). The CON received regular water, while other groups were administered a 0.05% NaI solution for 8 weeks to induce AIT pathology, validated by histopathological thyroid lymphocyte infiltration and elevated serum Thyroglobulin Antibody (TGAb) titers. Post-modeling, BZYQ mice were orally administered 9.56 g/kg herbal extract daily, the SYT group received 3.033×10^−5^ g/kg Selenium Yeast Tablet suspension, with CON and AIT groups maintained on water for 8 weeks. Following the 8-week experiment, mice were humanely euthanized under deep anesthesia (1% sodium pentobarbital, 50 mg/kg i.p.), with abolished pedal withdrawal and corneal reflexes confirming adequate anesthesia depth. Terminal blood collection *via* aseptic abdominal aorta puncture achieved controlled surgical exsanguination until circulatory collapse. Death confirmation preceded immediate post-mortem collection of thyroid glands and spleens.

### 2.4 Histological Examination (H&E)

Post-treatment, murine thyroid specimens were fixed in 4% paraformaldehyde, processed through graded ethanol dehydration, xylene clearing, and paraffin embedding. Microtome-generated 4μm sections were dewaxed with xylene, rehydrated *via* ethanol series, H&E-stained (C0105S, Beyotime Biotechnology, Shanghai, China), and mounted with neutral resin for microscopic evaluation at 400× magnification.

### 2.5 Determination of the Autoimmune Antibodies

Mouse serum was separated by centrifugation (1000 ×g, 10 min, rotor radius 10 cm) and analyzed using the commercial Enzyme-Linked Immunosorbent Assay (ELISA) kit (ml037680, ml002215, Shanghai Enzyme-linked Biotechnology Co., Ltd., Shanghai, China). Sample wells received 50 μL of undiluted serum. All wells, excluding blanks, were treated with 100 μL HRP-conjugated detection antibody and incubated at 37°C for 60 min. After five washing cycles, substrate solution was introduced, and plates were incubated at 37°C in the dark for 15 min. The reaction was terminated, and absorbance at 450 nm was measured. TGAb and Thyroid Peroxidase Antibody (TPOAb) concentrations were calculated based on the standard curve.

### 2.6 Determination of Lactate Concentration

Serum samples were diluted 5-fold, mixed with enzyme reagent and chromogenic agent following the protocol of the commercial kit (A019-2-1, Nanjing Jiancheng Bioengineering Institute, Nanjing, China), and incubated at 37°C for 10 min. After terminating the reaction, absorbance values were measured to determine lactate concentration.

### 2.7 Western Blot Analysis

Thyroid tissue proteins were isolated using radioimmunoprecipitation assay buffer (R0010, Solarbio Science & Technology Co., Ltd., Beijing, China), quantified *via* bicinchoninic acid assay kit (WB6501, NCM Biotech Co., Ltd., Guangzhou, China), and electrophoretically separated for subsequent analyses. The membranes were incubated overnight at 4°C with primary antibodies diluted in blocking buffer as follows: PTEN (1:1000, YT5752, ImmunoWay Biotechnology, Texas, USA), PI3K (1:1000, IPB0061, Taizhou Baijia Biotechnology Co., Ltd., Taizhou, China) and its phosphorylated form phosphorylated PI3K (p-PI3K) (1:1000, IPH0018, Taizhou Baijia Biotechnology Co., Ltd.), AKT (1:1000, SRP00438, Saier Biotech, Tianjin, China) and its phosphorylated form phosphorylated AKT (p-AKT) (1:1000, IPH0007, Taizhou Baijia Biotechnology Co., Ltd.), GLUT1(1:1000, YT1928, ImmunoWay Biotechnology), Hexokinase 2 (HK2) (1:2000, 22029-1-AP, Wuhan Sanying Biotechnology Co., Ltd., Wuhan, China), Pyruvate Kinase M2 Isoform (PKM2) (1:1000, YT3777, ImmunoWay Biotechnology), Lactate Dehydrogenase A (LDHA) (1:1000, IPB7876, Taizhou Baijia Biotechnology Co., Ltd.). The membranes were incubated with a goat anti-rabbit IgG (H&L)-HRP secondary antibody (1:20,000, bs-0295G-HRP, Bioss Antibodies, Beijing, China), followed by chemiluminescent detection. Band intensities were quantified using ImageJ.

### 2.8 Immunofluorescence Localization

The thyroid tissue sections underwent dewaxing, rehydration, and antigen retrieval. Following blocking, Incubation at 4°C overnight with Retinoic acid receptor-related orphan receptor gamma t (RORγt) (1:100, Bs-23109R, Bioss Biotechnology Co., Ltd.) and HK2 (1:200, 22029-1-AP, Wuhan Sanying Biotechnology Co., Ltd.) primary antibodies. Subsequently, Hrp-labeled goat anti-rabbit (1:2000, ab6808, Abcam plc, Cambridge, UK) was incubated with secondary antibody at room temperature for 40 minutes, 4',6-Diamidino-2-phenylindole (DAPI) staining, and fluorescence microscope imaging.

### 2.9 Flow Cytometry

Splenocytes were isolated from erythrocyte-lysed spleen homogenates. Th17 cells were analyzed with the Mouse Th17 Staining Kit (KTH217, Hangzhou Lianke Biotechnology, Hangzhou Lianke Biotechnology, China). Cells were stained with anti-mouse CD3e-FITC (AM003E01, Hangzhou Lianke Biotechnology) and CD4-PerCP-Cy5.5 (AM00407, Hangzhou Lianke Biotechnology) for 15 min at room temperature in the dark. Following fixation/permeabilization, intracellular IL-17A-PE (AM011704, Hangzhou Lianke Biotechnology) staining was performed under identical conditions. Data were acquired using the flow cytometer (BD Biosciences, San Jose, CA, USA).

### 2.10 Statistical Analysis

All experimental data were derived from three independent technical replicates to ensure methodological rigor. Statistical analysis was performed using GraphPad Prism 8 (GraphPad Software, San Diego, CA, USA). Normally distributed variables are presented as $\overset{-}{x}$±S, whereas non-normally distributed variables are expressed as median (interquartile range). Parametric tests (ANOVA) are applied to normally distributed data, while non-parametric alternatives addressed non-normal distributions or heteroscedastic datasets. Statistical significance is defined at *P* < 0.05.

## 3 RESULTS

### 3.1 BZYQ Alleviates the Histological Pathological Changes of the Thyroid

To evaluate BZYQ's therapeutic effects on thyroid histopathology, the study assessed thyroid tissues using H&E staining. Morphological analysis emphasized follicular architecture and inflammatory infiltration as key features. CON group thyroid tissues exhibited intact follicular architecture with tightly arranged epithelial cells and absent lymphocytic infiltration. In contrast, mice in the AIT group showed significant thyroid enlargement, characterized by dilated follicular lumens, structural disorganization, and substantial interstitial lymphocyte accumulation. Therapeutic intervention with BZYQ or SYT effectively reduced thyroid volume compared to AIT controls, accompanied by histological improvements: normalized follicular epithelial alignment, decreased lumen dilation, and attenuated lymphocyte infiltration (Figs. **1A** and **B**).

### 3.2 BZYQ reduces TGAb and TPOAb Levels

To evaluate BZYQ's immunomodulatory efficacy, thyroid autoantibodies were quantified *via* ELISA. TGAb/TPOAb levels in the AIT group serum markedly exceeded CON group values (TPOAb: *P* < 0.0001, 95% CI: -23.60–-19.22; TGAb: *P* < 0.0001, 95% CI: -3.755 to -2.271; Fig. **2A**-**B**). Compared to the AIT group, both BZYQ and SYT treatments significantly reduced TPOAb levels (BZYQ *vs* AIT: *P* < 0.0001, 95% CI: 15.54 to 19.92; SYT *vs* AIT: *P* < 0.0001, 95% CI: 10.49 to 14.88; Figs. **2A** and **B**). For TGAb, both BZYQ and SYT still effectively lowered TGAb levels relative to AIT mice (BZYQ *vs* AIT: *P* < 0.0001, 95% CI: 1.957 to 3.441; SYT *vs* AIT: *P* = 0.0005, 95% CI: 0.5919–2.076; Figs. **2A** and **B**).

### 3.3 BZYQ Reduces Lactate Levels

Serum lactate quantification demonstrated a pronounced increase in the AIT group relative to CON (*P* < 0.0001, 95% CI: -5.894 to -4.052; Fig. **3**). Both BZYQ and SYT interventions substantially attenuated lactate accumulation compared to untreated AIT mice (BZYQ *vs* AIT: *P* < 0.0001, 95% CI: 5.548 to 7.391; SYT *vs* AIT: *P* < 0.0001, 95% CI: 2.621 to 4.464; Fig. **3**).

### 3.4 BZYQ Regulates PTEN/AKT Signaling Pathway

To explore the potential mechanisms of BZYQ in improving autoimmunity and thyroid tissue inflammation, Western blot analysis was employed to examine the protein expression levels of the PTEN/AKT signaling pathway (Fig. **4A**). Compared with the CON group, the AIT group showed significantly decreased PTEN protein expression (*P* < 0.0001, 95% CI: 0.2695 to 0.5661; Fig. **4B**) in thyroid tissues, along with increased expression of p-PI3K/PI3K (*P* = 0.0004, 95% CI: -0.3864 to -0.1501; Fig. **4C**) and p-AKT/AKT (*P* < 0.0001, 95% CI: -0.6251 to -0.3876; Fig. **4D**). Compared to the AIT group, PTEN protein expression was significantly elevated in both treatment groups (BZYQ *vs * AIT: *P* = 0.001, 95% CI: -0.4433 to - 0.1467; SYT *vs* AIT: *P* = 0.0019, 95% CI: -0.4145 to -0.1179; Fig. **4B**); notably, both BZYQ and SYT treatments significantly reduced p-PI3K/PI3K levels (BZYQ *vs* AIT: *P* = 0.0168, 95% CI: 0.02912 to 0.2655; SYT *vs* AIT: *P* = 0.0429, 95% CI: 0.004012 to 0.2404; Fig. **4C**) and p-AKT/AKT levels (BZYQ *vs* AIT: *P* = 0.0031, 95% CI: 0.0794 to 0.317; SYT *vs* AIT: *P* = 0.0006, 95% CI: 0.1376 to 0.3752; Fig. **4D**).

### 3.5 BZYQ Inhibits Key Glycolytic Molecules

The therapeutic impact of BZYQ on AIT-associated immunometabolic imbalance was assessed through quantification of key glycolytic regulators, including the glucose transporter GLUT1 and rate-limiting enzymes (Fig. **5A**). Compared with the CON group, the AIT group exhibited significantly elevated expression levels of GLUT1 (*P* < 0.0001, 95% CI: -1.379 to -0.6857; Fig. **5B**), HK2 (*P* < 0.0001, 95% CI: -1.221 to -0.6223; Fig. **5C**), PKM2 (*P* < 0.0001, 95% CI: -1.252 to -0.6743; Fig. **5D**), and LDHA (*P* < 0.0001, 95% CI: -1.141 to -0.6820; Fig. **5E**). Conversely, both BZYQ and SYT treatments markedly downregulated these proteins compared to the AIT group: GLUT1 (BZYQ *vs* AIT: *P* = 0.0087, 95% CI: 0.1396 to 0.8329; SYT *vs* AIT: *P* = 0.0012, 95% CI: 0.3188 to 1.012; Fig. **5B**), HK2 (BZYQ *vs* AIT: *P* = 0.0287, 95% CI: 0.03715 to 0.6364; SYT *vs* AIT: *P* = 0.0046, 95% CI: 0.1684 to 0.7676; Fig. **5C**), PKM2 (BZYQ *vs* AIT: *P* = 0.0048, 95% CI: 0.1590 to 0.7368; SYT *vs* AIT: *P* = 0.0024, 95% CI: 0.2125 to 0.7903; Fig. **5D**), and LDHA (BZYQ *vs* AIT: *P* < 0.0001, 95% CI: 0.4404 to 0.8992; SYT *vs* AIT: *P* < 0.0001, 95% CI: 0.4214 to 0.8802; Fig. **5E**).

### 3.6 BZYQ Reduces the Localized Expression of HK2 and RORγt Proteins

The therapeutic effects of BZYQ on glycolysis and Th17 cell differentiation in AIT were further investigated through immunofluorescence staining. Thyroid tissue analysis focused on HK2, a glycolytic rate-limiting enzyme, and RORγt, the master transcriptional regulator of Th17 cells, to evaluate their spatial distribution and expression dynamics. Compared with the CON group, the AIT group exhibited significantly enhanced positive immunofluorescent signals for both HK2 and RORγt; Conversely, treatment with BZYQ resulted in marked attenuation of these signals in the group (Fig. **6**).

### 3.7 BZYQ Reduces the Proportion of Th17 Cells

Flow cytometric analysis was conducted to assess the inhibitory effects of BZYQ on Th17 cell differentiation abnormalities in the AIT model mice, with quantification of Th17 cell proportion changes. The results revealed that, compared with the CON group, the proportion of Th17 cells was significantly increased in the AIT group (*P* = 0.0001, 95% CI: -8.3000 to -3.7470; Figs. **7A**-**C**). Conversely, both BZYQ and SYT treatment groups exhibited a marked reduction in Th17 cell proportions compared to the AIT group (BZYQ *vs* AIT: *P* = 0.0009, 95% CI: 2.3470 to 6.9000; SYT *vs* AIT: *P* = 0.0097, 95% CI: 0.8600 to 5.413; Figs. **7A**-**C**).

### 3.8 Mechanism

Glucose enters cells *via* GLUT1 and is ultimately converted to lactate through the glycolytic pathway mediated by key enzymes including HK2, PKM2, and LDHA. Lactate upregulates RORγt expression, promoting Th17 cell differentiation and IL-17 secretion, thereby exacerbating inflammatory responses. As a negative regulator of the PI3K-AKT pathway, PTEN modulates glucose metabolism and inflammation by suppressing this signaling cascade (Fig. **8**).

## 4 DISCUSSION

BZYQ bioactive components regulate immunometabolic pathways, research shows. Liu *et al*. demonstrated *in vivo* that ginsenoside Rg3 alleviates aberrant glycolysis *via* activation of the PI3K/AKT signaling pathway [24]. Li Hongchang *et al*. reported that astragaloside IV suppresses glycolytic activity [25]. Previous studies have reported that Panax notoginseng saponins were shown to inhibit nuclear PKM2 expression, thereby reducing Th17 cells differentiation in collagen-induced arthritis models [26]. These findings collectively suggest that BZYQ may exert regulatory effects on Th17 cell differentiation through immunometabolic modulation.

Prior studies indicate that excessive iodine may induce marked thyroid injury and autoimmunity in mice through dysregulation of Th17 cells differentiation [27]. Among these mechanisms, CD4^+^ T cells differentiation-mediated immune damage serves as a critical pathogenic factor in AIT [28]. Th17 cells, identified as a subset of pro-inflammatory CD4^+^ T cells in 2005, are characterized by their dependence on RORγt ligands for differentiation, with IL-17 and Tumor necrosis factor-alpha (TNF-α) secretion mediating inflammation and autoimmunity [29]. Extensive evidence has established associations between Th17 cells and various autoimmune diseases, including rheumatoid arthritis and multiple sclerosis [30-32]. Wu *et al*. [33] reported that the percentage of Th17 cells in peripheral blood mononuclear cells (PBMCs) of AIT patients showed an iodine-dependent increase. In contrast, no significant changes were observed in healthy controls. Recent meta-analyses indicate elevated serum pro-inflammatory cytokines alongside reduced anti-inflammatory factors in HT patients. Systemic inflammatory imbalance promotes thyroid-localized Th17-polarized microenvironments *via* dual mechanisms [34]. Collectively, these data reveal that AIT progression involves Th17-skewed differentiation of CD4^+^ T cells, where metabolic reprogramming drives pathogenic conversion, aligning with established disease trajectories.

This study revealed that BZYQ treatment significantly downregulated the expression levels of GLUT1, HK2, PKM2, and LDHA in thyroid tissues, confirming its mechanism of action through immunometabolic reprogramming in AIT. Recent studies have highlighted the pivotal role of metabolic reprogramming in immune responses [35, 36]. The emerging concept of immunometabolism provides a framework to elucidate how dynamic metabolic adaptations in immune cells dictate their functional states across various diseases, ranging from cancer to chronic inflammation [37]. This theory posits that activated T cells enhance energy supply through metabolic pathway remodeling to meet the high energetic and biosynthetic demands of inflammation and autoimmunity. Different immune cells exhibit metabolic preferences, with proinflammatory Th17 cells relying on aerobic glycolysis (the Warburg effect) for energy production [38, 39]. The immunometabolic process of Th17 cells begins with GLUT1-mediated glucose uptake, followed by a 10-step aerobic glycolysis reaction catalyzed by key enzymes such as HK2 and PKM2 to produce pyruvate [40, 41]. Under sustained activation, LDHA converts pyruvate to lactate, a defining feature of aerobic glycolysis, and rapidly generates ATP to support Th17 cell differentiation [22, 42]. Lactate serves as a critical biomarker of metabolic pathway switching, and its elevated levels create an inductive microenvironment for Th17 differentiation [17]. Additionally, glycolytic enzymes have been shown to promote RORγt transcription, thereby driving Th17-mediated inflammatory responses [43]. Damasceno *et al*. [44] demonstrated that Th17 cell differentiation and pathogenicity in experimental autoimmune encephalomyelitis rely on metabolic reprogramming driving glycolytic transition, with PKM2 serving as a critical regulatory factor. Chen *et al*. [45] found that in primary Sjögren's syndrome, activated CD4^+^ T cells promote excessive production of IL-17A and other cytokines through enhanced aerobic glycolysis, and inhibition of glycolysis can reverse their dysfunction. Shen *et al*. [26] demonstrated that inhibiting nuclear PKM2 blocks Th17 cells differentiation in a rheumatoid arthritis model. Furthermore, LDHA-supported glycolytic ATP maintains a positive feedback loop through PI3K signaling, which is crucial for pathogenic Th17 responses [22]. Lei *et al*. [13] found that CD4^+^ T cells in Hashimoto’s thyroiditis (HT) patients exhibited significantly enhanced glycolytic activity compared to healthy individuals. This study identifies glycolytic pathways as central mediators of Th17 cell dysfunction, with Th17-driven metabolic reprogramming directly accelerating AIT progression. Integrated analysis of experimental and published data delineates metabolic determinants of autoimmune pathogenesis.

Experimental data indicate that BZYQ upregulates PTEN expression, leading to suppression of PI3K p85 subunit and AKT phosphorylation. By reducing GLUT1-mediated glucose uptake, this mechanism modulates immunometabolic reprogramming. Consequently, glycolysis is suppressed, leading to a decreased proportion of Th17 cells in AIT mouse models. The PI3K pathway functions as a critical intracellular signaling cascade, regulating diverse biological processes [46]. Accumulating evidence links PI3K pathway activation to the pathogenesis and progression of AIT [47]. As a phosphoinositide kinase localized within cells [48]. PI3K phosphorylates PIP2 to generate PIP3 upon activation, subsequently recruiting and activating AKT. This signaling axis is counteracted by PTEN through negative regulation, ensuring signal transduction fidelity [49]. GLUT1, a 12-transmembrane glucose transporter expressed on cell membranes, plays a central role in glucose transport dynamics [50]. During metabolic reprogramming toward glycolysis, GLUT1 translocates to the plasma membrane to facilitate glucose uptake, a process dependent on PI3K/AKT pathway activation [51].

Certain limitations are acknowledged in this study. Inherent physiological, metabolic, and immunological disparities between humans and animal models constrain direct extrapolation of pathophysiological mechanisms. Furthermore, the multi-target nature of herbal formulations necessitates preclinical dose optimization and rigorous human trials to confirm their therapeutic precision and safety.

## CONCLUSION

In summary, BZYQ exerts regulatory effects on the aberrant differentiation of Th17 cells in AIT under conditions of immunometabolic dysfunction, potentially through modulating the PTEN/AKT pathway-driven enzymatic dysregulation that suppresses glycolysis, thereby reducing lactate production abnormalities. This study provides a potential therapeutic strategy for restoring immune homeostasis in AIT during clinical treatment. Subsequent studies should further elucidate dynamic regulatory mechanisms of immunometabolism in AIT, refine the multi-target regulatory network of BZYQ, and accelerate clinical translation.

## Figures and Tables

**Fig. (1):**
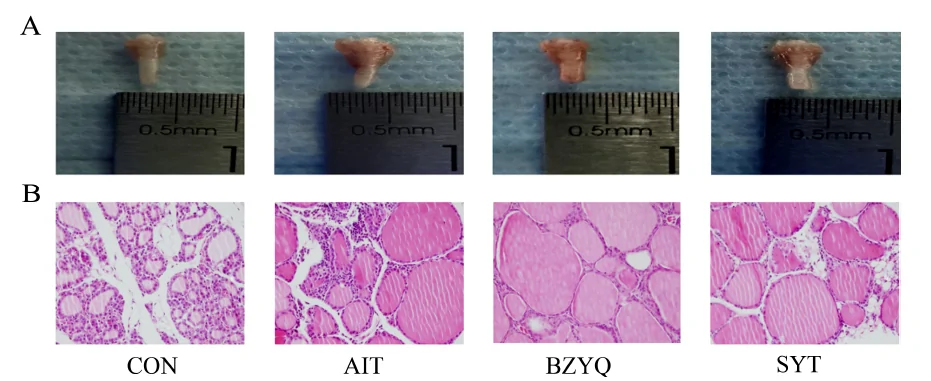
Effects of BZYQ on thyroid volume and inflammation in AIT mice. (**A**) Photographs showing changes in thyroid tissue volume; (**B**) Representative micrographs (×400) stained with H&E showing areas of follicular structural pathology and lymphocyte infiltration. Data are representative of three biologically independent experiments.

**Fig. (2):**
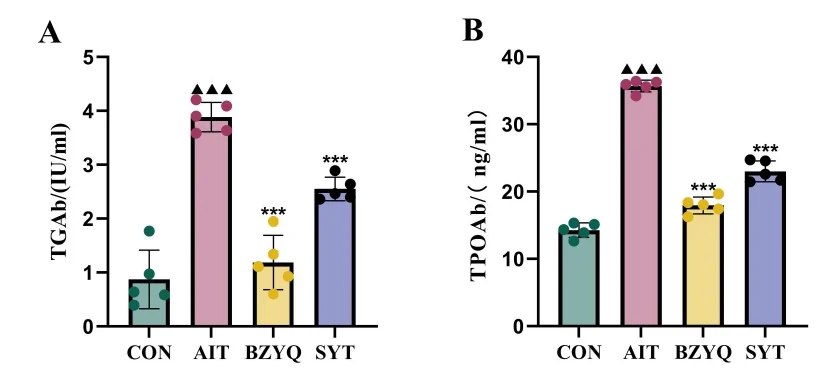
Effects of BZYQ on serum TGAb and TPOAb levels in AIT mice. (**A**) Serum TGAb level; (**B**) Serum TPOAb levels. *P*-value from ANOVA test. Compared with the CON group, ▲▲▲ *p* < 0.001 compared with the AIT group, ***P < 0.001. Data are representative of five biologically independent experiments.

**Fig. (3):**
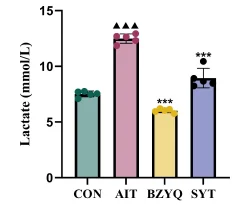
Effects of BZYQ on Lactate levels in serum. P-value from ANOVA test. Compared with the CON group, *p* < 0.001 compared with the AIT group, *P* < 0.001. Data are representative of five biologically independent experiments.

**Fig. (4):**
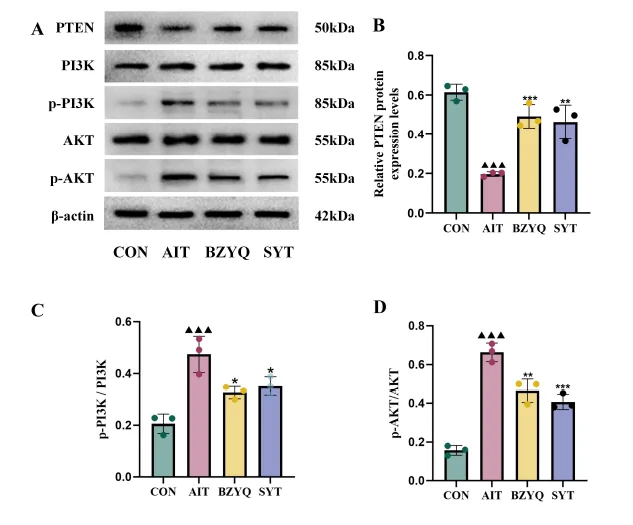
Effects of BZYQ on PTEN, PI3K, p-PI3K, AKT, and p-AKT proteins in thyroid tissue. (**A**) Representative western blot bands showing PTEN, PI3K, p-PI3K, AKT, p-AKT, and β-actin protein expressions in different treatment groups. (**B**) Ratio of PTEN to β-actin protein expression. (**C**) Ratio of p-PI3K to total PI3K protein expression. (**D**) Ratio of p-AKT to total AKT protein expression. *P*-value from ANOVA test. Compared with the CON group, ▲▲▲*P* < 0.001; compared with the AIT group, *P < 0.05, **P < 0.01,***P < 0.001. Data are representative of three biologically independent experiments.

**Fig. (5):**
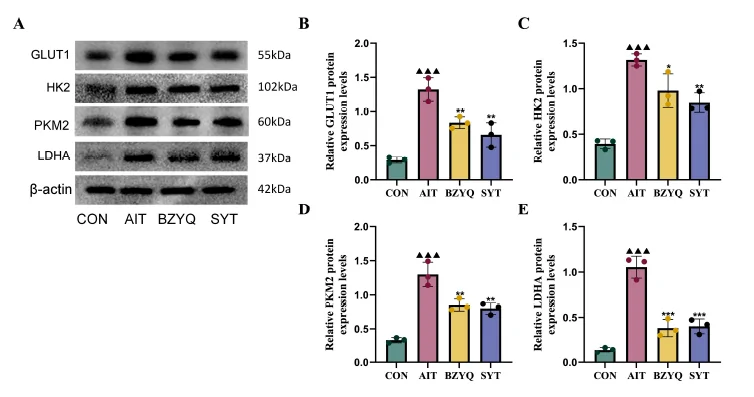
Effects of BZYQ on GLUT1, HK2, PKM2, and LDHA proteins in thyroid tissue. (**A**) Representative western blot bands showing GLUT1, HK2, PKM2, and LDHA protein expressions in different treatment groups. (**B**) Ratio of GLUT1 to β-actin protein expression. (**C**) Ratio of HK2 to β-actin protein expression. (**D**) Ratio of PKM2 to β-actin protein expression. (**E**) Ratio of LDHA to β-actin protein expression. P-value from ANOVA test. Compared with the CON group, ▲▲▲*P* < 0.001; compared with the AIT group, *P < 0.05, **P < 0.01,***P < 0.001. Data are representative of three biologically independent experiments.

**Fig. (6):**
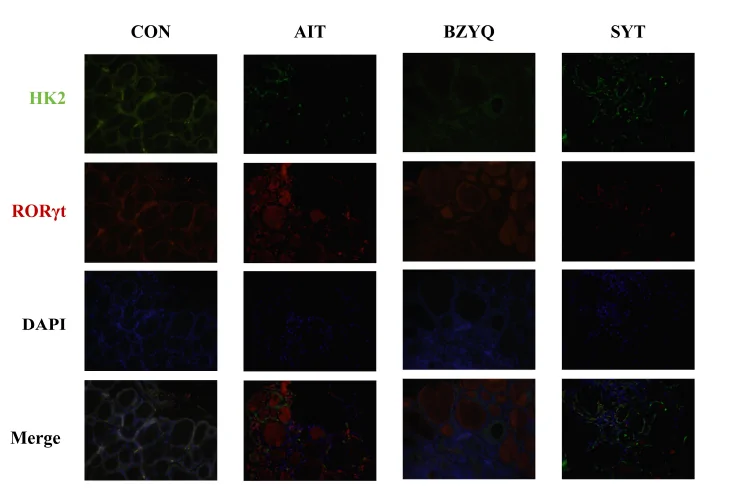
Effects of BZYQ on the localization and expression of HK2 and RORγt proteins. Immunofluorescence results of four groups (CON, AIT, BZYQ, SYT) show marker staining: HK2 (green), RORγt (red), DAPI (blue, nucleus). Merge showcases superimposed staining. Data are representative of three biologically independent experiments.

**Fig. (7):**
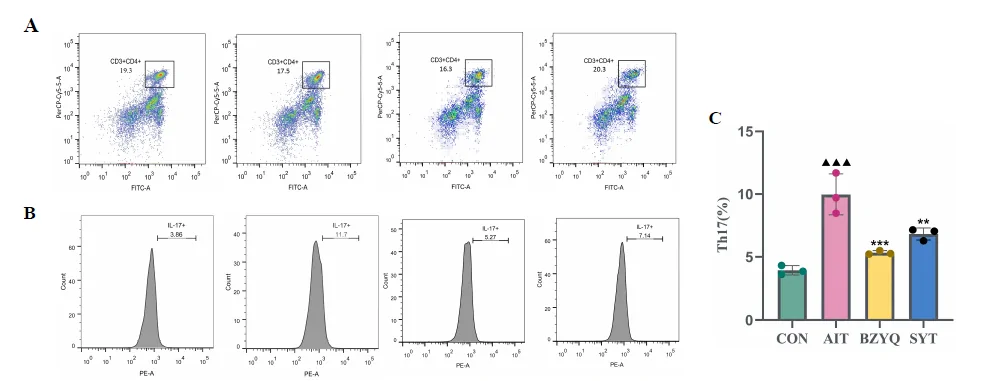
Effects of BZYQ on the proportion of Th17 cells. (**A**) Demonstrates the distribution of Th17 cells within CD3^+^CD4^+^T cells in CON, AIT, BZYQ, and SYT treatment groups. (**B**) Flow cytometry plots provide a detailed visualization of Th17 cell distribution. Representative gating strategy for Th17 cells (IL-17A^+^ within CD3^+^CD4^+^ T cells). (**C**) Quantification of the proportion of Th17 cells (Th17%). P-value from ANOVA test. Compared with the CON group, *P* < 0.001 compared with the AIT group, **P < 0.01, *P* < 0.001. Data are representative of three biologically independent experiments.

**Fig. (8):**
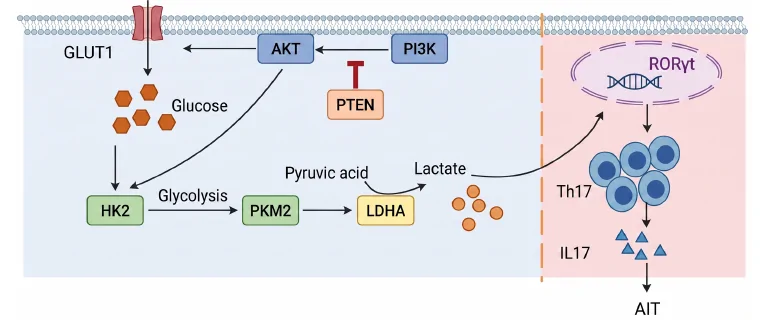
Schematic diagram of the mechanism.

## Data Availability

All data generated or analyzed during this study are included in this published article.

## LIST OF ABBREVIATIONS

AIT: Autoimmune Thyroiditis
AKT: Protein Kinase B
ANOVA: Analysis of Variance
ATP: Adenosine Triphosphate
BSA: Bovine Serum Albumin
BZYQ: Buzhong Yiqi Decoction
CON: Control Group
DAPI: 4',6-Diamidino-2-Phenylindole
EDTA: Ethylenediaminetetraacetic Acid
ELISA: Enzyme-Linked Immunosorbent Assay
GLUT1: Glucose Transporter 1
HE: Hematoxylin And Eosin
HK2: Hexokinase 2
HRP: Horseradish Peroxidase
IF: Immunofluorescence
IL-17: Interleukin-17
I.P.: Intraperitoneal
LDHA: Lactate Dehydrogenase A
NaI: Sodium Iodide
PBS: Phosphate-Buffered Saline
PI3K: Phosphatidylinositol 3-Kinase
PKM2: Pyruvate Kinase M2 Isoform
PTEN: Phosphatase And Tensin Homolog
RORγt: Retinoic Acid Receptor-Related Orphan Receptor Gamma T
SYT: Selenized Yeast Group
Th17: T Helper 17 Cell
TGAb: Thyroglobulin Antibody
TNF-α: Tumor Necrosis Factor-Alpha
TPOAb: Thyroid Peroxidase Antibody
